# Association between 16S rRNA gene mutations and susceptibility to amikacin in *Mycobacterium avium* Complex and *Mycobacterium abscessus* clinical isolates

**DOI:** 10.1038/s41598-021-85721-5

**Published:** 2021-03-17

**Authors:** Su-Young Kim, Dae Hun Kim, Seong Mi Moon, Ju Yeun Song, Hee Jae Huh, Nam Yong Lee, Sung Jae Shin, Won-Jung Koh, Byung Woo Jhun

**Affiliations:** 1 Division of Pulmonary and Critical Care Medicine, Department of Medicine, Samsung Medical Center, Sungkyunkwan University School of Medicine, Irwon-ro 81, Gangnam-gu, Seoul, 06351 South Korea; 2 Division of Pulmonary and Critical Care Medicine, Department of Medicine, Samsung Changwon Hospital, Sungkyunkwan University School of Medicine, Changwon, South Korea; 3 Department of Laboratory Medicine and Genetics, Samsung Medical Center, Sungkyunkwan University School of Medicine, Seoul, South Korea; 4 Department of Microbiology, Yonsei University College of Medicine, Seoul, South Korea; 5 Institute for Immunology and Immunological Disease, Yonsei University College of Medicine, Seoul, South Korea; 6 Brain Korea 21 Program for Leading Universities and Students (PLUS) Project for Medical Science, Yonsei University College of Medicine, Seoul, South Korea

**Keywords:** Microbiology, Antimicrobials

## Abstract

We evaluated the association between 16S rRNA gene (*rrs*) mutations and susceptibility in clinical isolates of amikacin-resistant nontuberculous mycobacteria (NTM) in NTM-pulmonary disease (PD) patients. Susceptibility was retested for 134 amikacin-resistant isolates (minimum inhibitory concentration [MIC] ≥ 64 µg/ml) from 86 patients. Amikacin resistance was reconfirmed in 102 NTM isolates from 62 patients with either *Mycobacterium avium* complex-PD (MAC-PD) (n = 54) or *M. abscessus*-PD (n = 8). MICs and *rrs* mutations were evaluated for 318 single colonies from these isolates. For the 54 MAC-PD patients, *rrs* mutations were present in 34 isolates (63%), comprising all 31 isolates with amikacin MICs ≥ 128 µg/ml, but only three of 23 isolates with an MIC = 64 µg/ml. For the eight *M. abscessus*-PD patients, all amikacin-resistant (MIC ≥ 64 µg/ml) isolates had *rrs* mutations. In amikacin-resistant isolates, the A1408G mutation (n = 29) was most common. Two novel mutations, C1496T and T1498A, were also identified. The culture conversion rate did not differ by amikacin MIC. Overall, all high-level and 13% (3/23) of low-level amikacin-resistant MAC isolates had *rrs* mutations whereas mutations were present in all amikacin-resistant *M. abscessus* isolates. These findings are valuable for managing MAC- and *M. abscessus*-PD and suggest the importance of phenotypic and genotypic susceptibility testing.

## Introduction

Nontuberculous mycobacteria (NTM) are ubiquitous organisms that cause chronic disease, and the burdens of NTM-pulmonary disease (PD) are increasing globally, including in South Korea^1,2^. Among NTM species, *Mycobacterium avium* complex (MAC), which is mainly composed of *M. avium* and *M. intracellulare*, is the most common pathogen; and *M. abscessus*, predominantly composed of *M. abscessus* subsp. *abscessus* and *M. abscessus* subsp. *massiliense*, is the second most common pathogen in many countries^3,4^.

Amikacin is one of the most important parenteral antibiotics for treating NTM-PD, especially for MAC-PD and *M. abscessus*-PD^5,6^. For MAC-PD, amikacin is recommended for patients with advanced disease or those whose isolates acquired macrolide resistance^7,8^. Evidence from the CONVERT study indicates a benefit of adding liposomal amikacin inhalation for refractory MAC-PD^9^. Recent guidelines from the American and British Thoracic Societies for treating *M. abscessus*-PD recommend a multidrug therapy, including intravenous amikacin, based on results of drug susceptibility testing (DST)^7,8,10^. A meta-analysis also showed that parenteral amikacin use was associated with *M. abscessus*-PD treatment success^11^. Unfortunately, treatment outcomes for NTM-PD are still unsatisfactory, and a substantial proportion of NTM-PD patients remain refractory to treatment^12–15^. Given that approximately 90% of MAC or *M. abscessus* clinical isolates have been reported to be amikacin susceptible^16,17^, amikacin is one of the few drugs that can be used as salvage therapy in refractory disease, and it plays an important role in long-term NTM-PD treatment.

Acquired resistance to amikacin results from point mutations at positions 1406, 1408, 1409, and 1491 in the *rrs* gene, encoding 16S rRNA^18,19^, and isolates with these mutations are mostly isolated from patients with extensive exposure to amikacin or related aminoglycosides^20,21^. However, there are limited data from NTM clinical isolates on the association between these mutations and amikacin susceptibility, or on the clinical outcomes of patients with amikacin-resistant NTM-PD. The purposes of this study were to elucidate the association between *rrs* mutations and amikacin resistance in amikacin-resistant MAC and *M. abscessus* clinical isolates and to evaluate the clinical outcomes of patients with amikacin-resistant NTM-PD according to the MIC of amikacin.

## Results

### DST for amikacin

**Figure 1:**
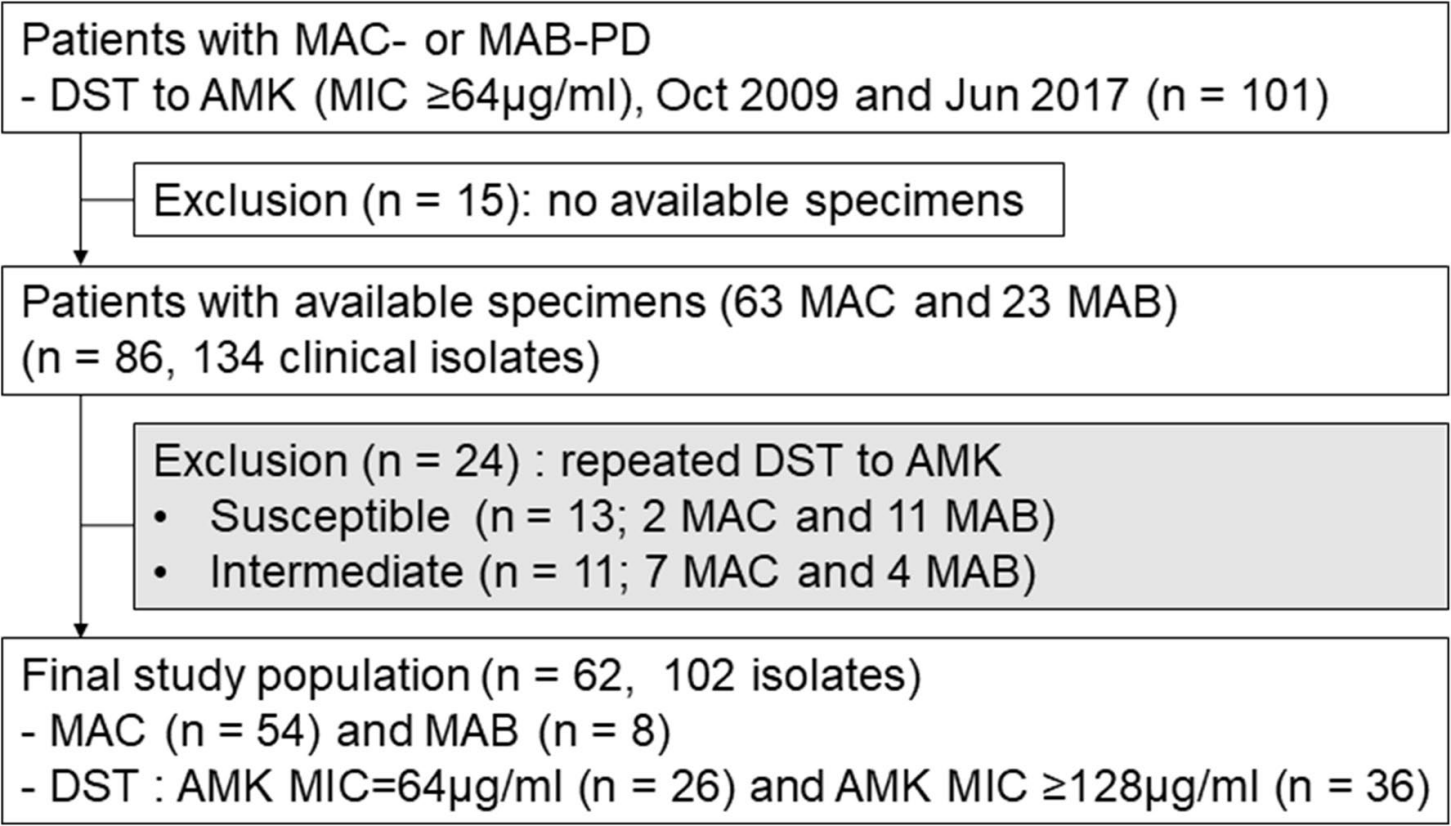
Study population. MAC, *M. avium* complex; MAB-PD, *M. abscessus*-pulmonary disease; DST, drug susceptibility testing; AMK, amikacin; MIC, minimum inhibitory concentration.

A total of 62 patients with MAC-PD (n = 54) or *M. abscessus*-PD (n = 8) were reconfirmed to have amikacin-resistant NTM isolates. As multiple isolates had been stored for some of these patients, a total of 102 isolates were available for analysis (Fig. 1), with at least one per patient. However, to avoid potential problems arising from any polyclonal infections, individual colonies were obtained for each isolate, and sometimes several colonies per isolate, yielding a total 318 single colonies for further analysis. Amikacin MICs for the single colonies are shown in Supplementary Table S1. If any of the single colonies obtained from an isolate had an amikacin MIC ≥ 64 µg/ml, the isolate was considered resistant to amikacin.

### Patient characteristics

The clinical characteristics of the 62 NTM-PD patients with amikacin-resistant isolates are shown in Table 1. The median age was 64 years (interquartile range [IQR], 53–70 years), and 28 (45%) were female. All but four patients had a history of exposure to amikacin or other aminoglycoside prior to confirmation of amikacin resistance. The median exposure duration was 91 days (IQR, 28–172 days). Most patients (87%) were infected with MAC, whereas eight (13%) were infected with *M. abscessus*.

**Table 1:** Clinical characteristics of the 62 patients with NTM-PD^a^.

Characteristic	Total (n = 62)
Age, years	64 (53–70)
Female	28 (45)
**Exposure to amikacin**	
Intravenous administration^b^	23 (37)
Inhaled administration	1 (2)
Other aminoglycoside (streptomycin, kanamycin)	34 (55)
Unknown	4 (6)
Duration of amikacin exposure (days)	91 (28–172)
**Etiology**	
MAC	54 (87)
* M. avium*	21/54
* M. intracellulare*	32/54
* M. chimaera*	1/54
*M. abscessus*	8 (13)
* M. abscessus* subsp. *abscessus*	5/8
* M. abscessus* subsp. *massiliense*	3/8

*NTM-PD* nontuberculous mycobacterial pulmonary disease, *MAC M. avium* complex.

^a^Values are numbers (%) or medians (interquartile ranges).

^b^Five patients were exposed to both amikacin and another aminoglycoside.

### *rrs* mutations

Supplementary Table S1 shows the results of *rrs* sequence analysis of the 318 single colonies obtained from the 102 stored clinical isolates, which included 84 MAC isolates and 18 M*. abscessus* isolates. There were 250 single colonies from the 84 MAC isolates, and of these, all 50 colonies with an MIC ≥ 128 µg/ml had *rrs* mutations. However, of the 134 single colonies with an amikacin MIC = 64 µg/ml, only three colonies (from patients no. A-7, A-16, and A-19 in Supplementary Table S1) had *rrs* mutations. From the 18 M*. abscessus* isolates, there were 68 single colonies, and *rrs* mutations were present in all 42 of them that had an amikacin MIC ≥ 64 µg/ml. None of the single colonies from the MAC or *M. abscessus* isolates with susceptible or intermediate susceptibility to amikacin (≤ 32 µg/ml) had *rrs* mutations.

Our analyses were summarized based on isolate susceptibility to amikacin and *rrs* mutations, using a single representative isolate for each patient (Table 2). For patients who had two or more stored isolates, if any single colony had an *rrs* mutation, the isolate from which that mutant colony was obtained was chosen as the representative isolate for that patient. Consequently, among the 54 patients with MAC-PD, there were 31 patients who had representative isolates with an amikacin MIC ≥ 128 µg/ml, all of which had *rrs* mutations. However, of the remaining 23 MAC-PD patients, i.e., those with isolates with an amikacin MIC = 64 µg/ml, only three had representative isolates with *rrs* mutations. For the eight patients with *M. abscessus*-PD, all amikacin-resistant isolates had *rrs* mutations. Overall, 42 patients (34/54 with MAC-PD and 8/8 with *M. abscessus*-PD) developed amikacin-resistant disease due to *rrs* mutations.

**Table 2:** Amikacin susceptibility and *rrs* mutations in MAC and *M. abscessus* clinical isolates.

Species	Amikacin MIC (µg/ml)	No. of isolates	No. of isolates with *rrs* mutation at the specified positions^a^						
			None	A1408G	C1409T	G1491C	G1491T	C1496T	T1498A
MAC (n = 54)	64	23	20	1	0	1	0	1	0
	128	5	0	3	0	1	0	1	0
	256	3	0	0	2	0	1	0	0
	> 256	23	0	21	0	1	1	0	0
*M. abscessus* (n = 8)	64	3	0	0	3	0	0	0	0
	128	0	0	0	0	0	0	0	0
	256	1	0	0	0	0	0	0	1
	> 256	4	0	4	0	0	0	0	0
Total		62	20	29	5	3	2	2	1

*MAC M. avium* complex, *MIC* minimum inhibitory concentration.

^a^*Escherichia coli* numbering.

**Figure 2:**
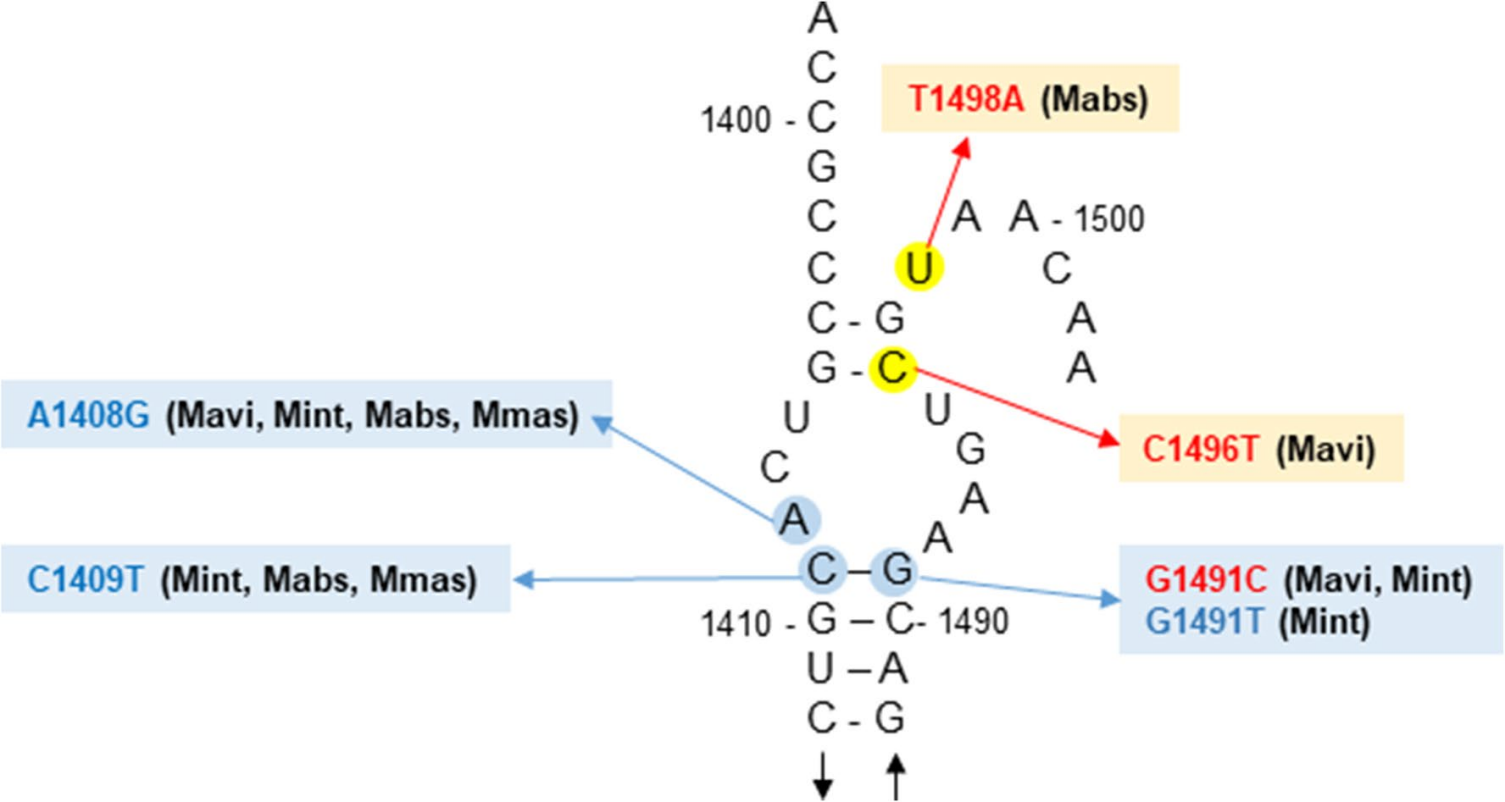
Locations of *rrs* mutations in amikacin-resistant MAC and *M. abscessus* clinical isolates. The species containing a given mutation are indicated with three-letter abbreviations: Mavi, *M. avium*; Mint, *M. intracellulare*; Mabs, *M. abscessus* subsp. *abscessus*; and Mmas, *M. abscessus* subsp. *massiliense*. *E. coli* numbering is used for the mycobacterial *rrs* sequence, and nucleotide positions at which mutations confer amikacin resistance are marked with blue or yellow circles. Blue circles indicate *rrs* mutations previously found in *M. tuberculosis* clinical isolates and *M. abscessus* subsp. *abscessus* mutants selected in vitro, as well as in this study. Yellow circles indicate novel mutations identified in this study.

Of the 42 representative isolates with *rrs* mutations, 29 (69%) harbored a mutation of A to G at position 1408 (A1408G). Five had a C1409T mutation, three had a G1491C mutation, and two had a G1491T mutation. Two novel mutations were identified; two *M. avium* isolates had a C1496T mutation, and one *M. abscessus* subsp. *abscessus* isolate had a T1498A mutation. A secondary structure model indicating the location of the *rrs* mutations in the MAC and *M. abscessus* clinical isolates is shown in Fig. 2.

### Clinical outcomes of NTM-PD patients according to the MIC of amikacin

Overall, of the 62 study patients, 19 (31%) (13 with *M. avium*-PD and six with *M. intracellulare*-PD) achieved culture conversion, and the median time from treatment to culture conversion was 104 days (IQR, 35–270 days). Additionally, this culture conversion was achieved in 10 of 26 patients (39%) with MAC isolates with an amikacin MIC = 64 µg/ml and in nine of 36 patients (25%) with MAC isolates with an MIC ≥ 128 µg/ml (p = 0.279). No patients with *M. abscessus*-PD achieved culture conversion.

For the 19 patients (nine *M. avium-*PD, five *M. intracellulare*-PD, three *M. abscessus* subsp. *abscessus*-PD, and two *M. abscessus* subsp. *massiliense*-PD) who maintained amikacin therapy for ≥ 28 days (median 256 days, IQR 60–516 days) even after amikacin resistance was confirmed, the clinical outcome was re-analyzed. Among them, one patient with *M. intracellulare*-PD, out of 5 (20%) patients who had MAC or *M. abscessus* isolates with an MIC = 64 µg/ml, achieved culture conversion; and one patient with *M. avium*-PD, out of 14 (7%) patients who had MAC or *M. abscessus* isolates with an MIC ≥ 128 µg/ml, achieved culture conversion (p = 0.468). There was no statistical significance in the culture conversion rate according to amikacin MIC.

## Discussion

In this study, we showed that mutations in the 16S rRNA gene of NTM clinical isolates are associated with amikacin resistance, and we also identified two novel amikacin resistance-associated mutations. Notably, all amikacin-resistant *M. abscessus* isolates had *rrs* mutations. In MAC isolates, all isolates showing relatively high resistance (MIC ≥ 128 µg/ml) had *rrs* mutations, but only 13% (3/23) of isolates showing relatively low resistance (MIC = 64 µg/ml) had *rrs* mutations. Additionally, among MAC-PD patients who maintained amikacin therapy, the culture conversion rate was low in the relatively high MIC group, although not significantly so. However, a larger study with more patients may determine whether there are indeed significant differences between these two groups.

During amikacin therapy for NTM-PD, a susceptible strain can develop resistance. The mechanism of resistance in MAC isolates with high-level resistance and in *M. abscessus* isolates appears to be via *rrs* mutations, with the A1408G mutation being most common. The *rrs* mutations A1408G, C1409T, and G1491T had previously been found in kanamycin-resistant *M. tuberculosis* clinical isolates^22^, and these three mutations were also found in aminoglycoside-resistant *M. abscessus* subsp. *abscessus* mutants selected in vitro^19^. To our knowledge, ours is the first study to identify the G1491T mutation in MAC clinical isolates. Indeed, the mutation at G1491C and the additional two novel mutations, at C1496T and T1498A, which are also potentially involved in amikacin resistance, were first identified in mycobacterial isolates (Fig. 2). Based on our results, mechanisms of high resistance to amikacin involve *rrs* mutations.

However, these above-mentioned mutations were not present in all of our amikacin-resistant MAC isolates, and other mechanisms may contribute to low-level amikacin-resistance. In mycobacteria, aminoglycoside acetyltransferases are associated with low-level aminoglycoside resistance. The aminoglycoside acetyltransferase gene *aac*(*2′*) was identified as a determinant of weak aminoglycoside resistance in *M. fortuitum*^23^. Also, the enhanced intracellular survival (Eis) protein, an aminoglycoside acetyltransferase, which was originally identified as a virulence factor in *M. tuberculosis*, confers kanamycin resistance^24,25^. In *M. tuberculosis* clinical isolates, *rrs* mutations correlated with high-level amikacin and kanamycin resistance, and *eis* promoter mutations correlated with low-level amikacin and moderate-level kanamycin resistance^26^. In another study with multidrug-resistant *M. tuberculosis* isolates, *rrs* mutations were associated with high-level cross-resistance to amikacin and kanamycin, whereas *eis* promoter mutations were associated with kanamycin resistance but not amikacin resistance^27^. Aminoglycoside acetyltransferase activity could not be detected in MAC lysates in another study^28^, and thus, the role of these enzymes in MAC amikacin resistance is uncertain. Another possible drug-resistance mechanism is the drug efflux pump. The multidrug-resistant *M. tuberculosis* clinical isolate M7, which was additionally resistant to amikacin, had no *rrs* mutations but overexpressed *pstB*, an efflux pump gene^29^. Two efflux pump genes were also overexpressed in the amikacin- and kanamycin-resistant *M. tuberculosis* clinical isolate MT433, which lacked mutations, but overexpression of these genes in *M. tuberculosis* H37Ra strain did not increase the MIC against amikacin^30^. However, overexpression of *eis* in *M. tuberculosis* isolate MT433 was detected, suggesting that *eis* might be associated with kanamycin resistance^30^. There are limited studies about the basis for low-level amikacin resistance in *M. tuberculosis*, and further studies are also needed to understand the mechanism of low-level amikacin resistance in MAC.

In the process of selecting research subjects for our study, we identified cases where the amikacin MIC value in NTM clinical isolates changed across repeated measurements. The proportion of resistant isolates with both initial and repeated amikacin MICs ≥ 64 µg/ml was 72% (62/86, Fig. 1). Discrepancies in the amikacin MIC between initial and repeat testing of MAC isolates were also reported in a previous study; five of eight isolates with an initial amikacin MIC = 64 µg/ml had an MIC = 32 µg/ml on repeat testing, and seven of 10 isolates with an initial MIC > 64 µg/ml had reproducible results on repeat testing^20^. This phenomenon is thought to be due to polyclonal infection with strains with different amikacin MICs, but further studies are needed to elucidate any association. Nevertheless, repeat testing to confirm amikacin resistance may be helpful for determining therapeutic strategies for NTM-PD, especially MAC-PD.

In our study, amikacin MIC readings based on visible growth in the wells were uncertain for some MAC isolates, unlike for *M. abscessus* isolates. For MAC isolates with *rrs* mutations, all isolates had amikacin MICs ≥ 64 µg/ml, and two-thirds (26/34) had amikacin MICs > 256 µg/ml. The growth of these isolates was evidenced by cell deposits at the bottom of the well (Supplementary Fig. S1B), and thus amikacin MIC interpretation was straightforward. In contrast, the cell deposits for MAC isolates without *rrs* mutations appeared to grow sporadically in wells at amikacin concentrations ≥ 64 µg/ml (Supplementary Fig. S1A). Because amikacin MIC interpretation for these cases was uncertain, growth in 64 µg/ml wells was confirmed by inoculating from MIC cultures into antimicrobial-free solid media. In this study, 46 MIC cultures showed non-confluent growth in the 64, 128, or 256 µg/ml wells, and thus the cultures in the 64 µg/ml wells were inoculated onto solid media and incubated; 12 grew on solid media whereas 34 showed no growth (final amikacin MIC = 64 µg/ml). Therefore, unlike *M. abscessus*, interpretation of amikacin MICs in MAC isolates requires caution because some strains appear to grow sporadically. Repeat DST for amikacin and analysis for *rrs* mutations can be helpful for appropriate treatment.

From a clinical perspective, although it was not statistically significant, the negative culture conversion rate in our data tended to be slightly higher in patients with isolates with MIC = 64 µg/ml than in patients with MIC ≥ 128 µg/ml. According to the Clinical and Laboratory Standards Institute (CLSI), 64 µg/ml represents a resistance breakpoint to amikacin. Griffith and colleagues reported that treatment success correlates with the following amikacin MIC breakpoints: susceptible, ≤ 64 µg/ml; and resistant, > 64 µg/ml^9,31^. The latest CLSI guidelines included two separate sets of AMK breakpoints for MAC as follows: (1) susceptible, ≤ 16 μg/ml; intermediate, 32 μg/ml; and resistant, ≥ 64 μg/ml, for intravenous AMK; and (2) susceptible, ≤ 64 μg/ml; and resistant, ≥ 128 μg/ml, for liposomal inhaled AMK^32^. In our current study, of the 62 MAC- and *M. abscessus*-PD patients with amikacin-resistant isolates, all but four patients had a history of exposure to amikacin or other aminoglycoside prior to confirmation of amikacin resistance. We maintained amikacin in our study, even when amikacin resistance was confirmed, to obtain the synergistic effects with clofazimine or when resistance results were confirmed relatively late. In a recent CONVERT study that showed efficacy of liposomal amikacin inhalation for refractory MAC-PD, approximately 34% of patients (10/29) having MAC isolates with an MIC = 64 µg/ml achieved culture conversion^9^, which suggests that amikacin may have some beneficial effects against MAC with low amikacin resistance; however, there is still no direct evidence for such benefits, and further studies on the association between MIC level and clinical outcomes are needed.

In conclusion, we identified several novel *rrs* mutations associated with amikacin resistance and determined that all high-level amikacin-resistant isolates and 13% of low-level amikacin-resistant isolates of MAC had *rrs* mutations whereas mutations were present in all amikacin-resistant *M. abscessus* isolates. Notably, there were some discrepancies between initial and retested susceptibility, suggesting that repeat phenotypic and genotypic susceptibility testing may be helpful. Finally, clinical outcomes were poor after the development of amikacin resistance in patients with MAC-PD or *M. abscessus*-PD. Overall, these findings should aid in our understanding of amikacin resistance in MAC- and *M. abscessus*-PD and its importance in patient management.

## Methods

### Study population

The NTM Registry of the Samsung Medical Center in South Korea (ClinicalTrials.gov identifier: NCT00970801) was screened for patients with MAC- or *M. abscessus*-PD who had DST data obtained between October 2009 and June 2017 and which showed an amikacin MIC ≥ 64 µg/ml, resulting in a total of 101 patients. All patients fulfilled the diagnostic criteria for NTM-PD^7^ and were enrolled in an Institutional Review Board-approved observational cohort study investigating NTM-PD at Samsung Medical Center (approval no. 2008-09-016). Informed consent was obtained from all individual participants, and this study was approved by an Institutional Review Board at Samsung Medical Center. All methods were performed in accordance with the relevant guidelines and regulations. After excluding 15 patients without stored isolates, 86 NTM-PD patients were initially examined. A total of 134 amikacin-resistant NTM clinical isolates from the 86 NTM-PD patients (one to six isolates per patient) were retested for amikacin susceptibility. Twenty-four patients who had only susceptible or intermediately resistant isolates on repeat testing were further excluded (nine MAC and 15 *M. abscessus*). Finally, 62 patients (54 MAC and eight *M. abscess*us) whose isolates had MICs ≥ 64 µg/ml on both initial and repeat testing were included, and 102 isolates were obtained and analyzed (Fig. 1).

### Microbiological examinations

In our hospital, processed specimens were inoculated into the BACTEC MGIT system (BD Diagnostics, Sparks, MD, USA). Liquid cultures were used for NTM identification and DST. NTM species were identified using PCR-restriction fragment length polymorphism analysis or reverse-blot hybridization of the *rpoB* gene in routine clinical practice^33^. Beginning in June 2014, species identification was conducted via nested multiplex PCR and a reverse-hybridization assay of the internal transcribed spacer (ITS) region (AdvanSure Mycobacteria GenoBlot Assay; LG Life Sciences, Seoul, South Korea)^34^. DST for amikacin was initially performed at the Korean Institute of Tuberculosis using the broth microdilution method^35^. For research purposes, NTM isolates from broth medium were first subcultured on 3% Ogawa agar slants. The whole slant cultures were then harvested and stored at − 80 °C.

From the 86 NTM-PD patients with available stored specimens (Fig. 1), a total of 134 NTM clinical isolates were propagated on Middlebrook 7H10 agar plates (Difco Laboratories, Detroit, MI, USA) supplemented with 10% (vol/vol) oleic acid-albumin-dextrose-catalase (OADC) (BD Diagnostics), and 425 single colonies were obtained. Three single colonies were routinely selected for each stored isolate or one to three single colonies of each morphotype when isolates included both smooth and rough morphotypes. Single colonies were re-identified using multilocus sequence analysis of *rrs*, *hsp*65, the 16S-23S rRNA gene ITS, and *rpoB*^36,37^ and were retested for amikacin susceptibility in the laboratory using the same method^35^. MAC and *M. abscessus* isolates were considered susceptible (MIC ≤ 16 µg/ml), intermediate (MIC = 32 µg/ml), or resistant (MIC ≥ 64 µg/ml) to amikacin according to the CLSI M62 protocol^32^. For MAC isolates with non-confluent growth at the bottom of the MIC-testing wells at amikacin concentrations of ≥ 64 µg/ml, the growth in the 64 µg/ml wells was confirmed by inoculating from the MIC-testing cultures onto antimicrobial-free solid media (7H10 agar plates supplemented with OADC).

### Treatment and clinical outcomes

All patients were treated with macrolide-based multidrug regimens^38^. In MAC-PD patients, a macrolide-based regimen that included ethambutol and rifampin was used. Aminoglycosides were administered in patients with severe disease. In *M. abscessus*-PD patients, an initial 2- to 4-week course of amikacin and imipenem (or cefoxitin) was administered during hospitalization, together with oral medications including a macrolide or fluoroquinolone, after which oral regimens were maintained. Some patients with refractory NTM-PD received oral clofazimine or inhaled amikacin. Sputum culture conversion was defined as three consecutive negative cultures, and the time to culture conversion was defined as the time to the date of the first negative culture.

### *rrs* gene sequencing

Mycobacterial DNA was extracted using the DNeasy UltraClean Microbial Kit (Qiagen, Hilden, Germany) and was used as the template for PCR amplification of the last 351 bp of the *rrs* gene, from positions 1154–1504, using primers *rrs*1-F (5′-ATG ACG TCA AGT CAT CAT GCC-3′) and *rrs*1-R (5′-AGG TGA TCC AGC CGC ACC TTC-3′)^19^. The PCR products were purified and sequenced using the *rrs*1-F primer. The *rrs* sequences from clinical isolates were compared against the GenBank database by BLAST analysis (http://www.ncbi.nlm.nih.gov) and were analyzed by the CLUSTAL-W multiple sequence alignment program^39^ against corresponding sequences of *M. avium* subsp. *hominissuis* 104, *M. avium* subsp. *hominissuis* TH135, *M. intracellulare* ATCC 13950, *M. abscessus* subsp. *abscessus* ATCC 19977, and *M. abscessus* subsp. *massiliense* JCM 15300.

### Statistical analysis

Data are presented as number (%) for categorical variables and median (IQR) for continuous variables. Categorical variables were compared using the Pearson chi-square test or Fisher’s exact test. Statistical analyses were performed using the PASW software program (ver. 18.0; SPSS Inc., Chicago, IL, USA).

## Supplementary Information

Supplementary Figure S1.

Supplementary Table S1.
