# Supplementary figures and images for: Association between 16S rRNA gene mutations and susceptibility to amikacin in Mycobacterium avium Complex and Mycobacterium abscessus clinical isolates
Source: Sci Rep. 2021 Mar 17;11:6108. doi: 10.1038/s41598-021-85721-5 (PMC7969740; doi:10.1038/s41598-021-85721-5)

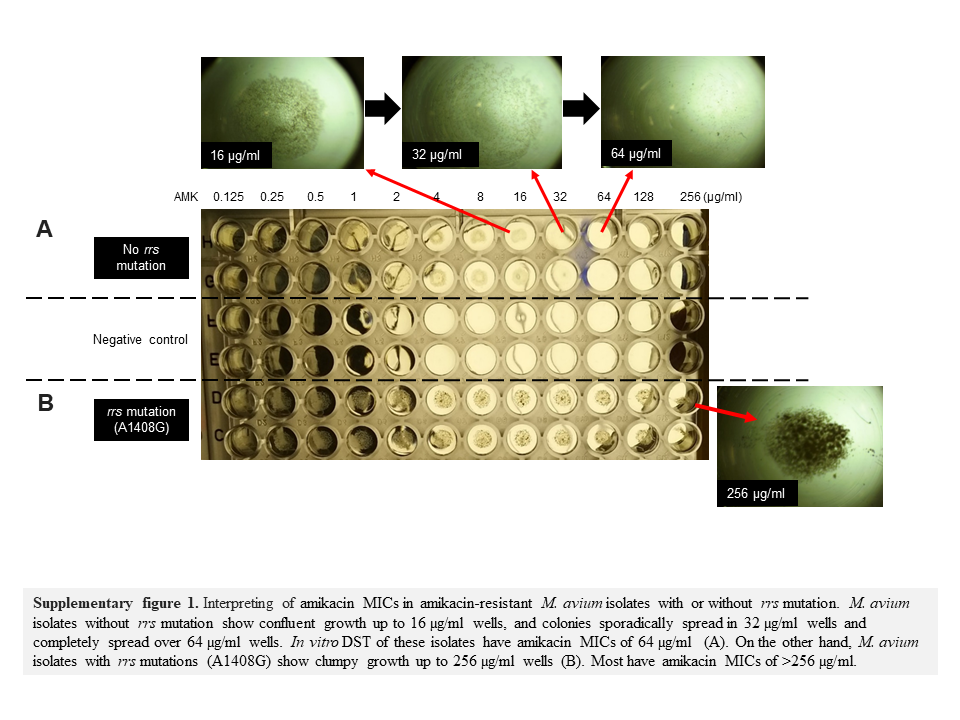

Supplement: Supplementary file 1 — Supplementary Figure S1. [file 41598_2021_85721_MOESM1_ESM.tif]
