# Supplementary material for: Association between 16S rRNA gene mutations and susceptibility to amikacin in Mycobacterium avium Complex and Mycobacterium abscessus clinical isolates
Source: Sci Rep. 2021 Mar 17;11:6108. doi: 10.1038/s41598-021-85721-5 (PMC7969740; doi:10.1038/s41598-021-85721-5)
Supplement: Supplementary file 2 — Supplementary Table S1. [file 41598_2021_85721_MOESM2_ESM.docx]

**Association Between 16S rRNA Gene Mutations and Susceptibility to Amikacin**

**in *Mycobacterium avium* Complex and *Mycobacterium abscessus* Clinical Isolates**

Su-Young Kim, Dae Hun Kim, Seong Mi Moon, Ju Yeun Song, Hee Jae Huh,

Nam Yong Lee, Sung Jae Shin, Won-Jung Koh, and Byung Woo Jhun

**Supplementary Table S1.** Analyses of 16S rRNA gene mutations in clinical isolates from 62 patients with initial and repeated AMK MICs ≥64 μg/ml

| Patient no. | Etiology | Exposure to AMK or other aminoglycosides (duration of exposure months) | Date of isolate | Isolate^*^ | Initial AMK MIC (μg/ml) | Colony morphotype | AMK MIC (μg/ml) in single colonies | *rrs* mutation in single colonies |
| --- | --- | --- | --- | --- | --- | --- | --- | --- |
| A-1 | *M. avium* | SM (1.9) | 10/28/2010 | A-1-1 | 64 | Smooth | 64, >256, 64 | WT, G1491C, WT |
|  |  |  | 05/09/2011 | A-1-2 | 64 | Smooth | 64, 64, 64 | WT, WT, WT |
| A-2 | *M. avium* | SM (4.3) | 11/30/2010 | A-2-1 | 64 | Smooth | 32, 32, 32 | WT, WT, WT |
|  |  | AMK (0.9) | 05/13/2011 | A-2-2 | 64 | Smooth | 32, 32, 32 | WT, WT, WT |
|  |  |  | 03/10/2012 | A-2-3 | 64 | Smooth | 64, 32, 64 | WT, WT, WT |
| A-3 | *M. avium* | AMK (0.5) | 03/07/2013 | A-3-1 | 64 | Smooth | 256, 16, >256 | C1409T, WT, A1408G |
| A-5 | *M. avium* | SM (3.8) | 04/17/2012 | A-5-1 | 64 | Smooth | 32, 64, 64 | NA, NA, WT |
| A-6 | *M. avium* | SM (2.6) | 08/23/2010 | A-6-1 | 128 | Smooth | 64, 64, >256 | WT, WT, A1408G |
|  |  |  | 08/30/2011 | A-6-2 | 64 | Smooth | 32, 32, 32 | WT, WT, WT |
|  |  |  | 04/10/2012 | A-6-3 | 64 | Smooth | 64, 64, 32 | WT, WT, WT |
| A-7 | *M. avium* | SM (3.5) | 01/07/2013 | A-7-1 | 64 | Smooth | 32, 64, 64 | WT, WT, G1491C |
| A-9 | *M. avium* | AMK (0.9) | 04/21/2014 | A-9-1 | 64 | Rough | 32, 32, 32 | WT, WT, WT |
|  |  |  | 11/29/2016 | A-9-2 | 64 | Smooth | 64, 64, 64 | WT, WT, WT |
| A-10 | *M. avium* | AMK (0.5), SM (9.8) | 05/19/2016 | A-10-1 | 128 | Smooth | >256, 256, >256 | A1408G, A1408G, A1408G |
| A-12 | *M. avium* | AMK (0.5) | 02/05/2013 | A-12-1 | 64 | Smooth | 64, 64, >256 | WT, WT, A1408G |
| A-13 | *M. avium* | SM (1.6) | 04/16/2013 | A-13-1 | 64 | Smooth | 16, 64, 64 | WT, WT, WT |
| A-14 | *M. avium* | KM (10.5) | 02/06/2013 | A-14-1 | 64 | Smooth | 64, 64, 64 | WT, WT, WT |
| A-15 | *M. avium* | AMK (0.5) | 09/20/2012 | A-15-1 | 64 | Smooth | 64, 64, 64 | WT, WT, WT |
| A-16 | *M. avium* | SM (3.0) | 12/03/2010 | A-16-1 | 64 | Smooth | 64, 64, >256 | WT, A1408G, A1408G |
| A-17 | *M. avium* | AMK (0.7), SM (3.1) | 10/30/2010 | A-17-1 | 64 | Smooth | >256, 64, >256 | A1408G, WT, A1408G |
| A-19 | *M. avium* | SM (3.7) | 01/19/2010 | A-19-1 | 128 | Smooth | 64, 64, 32 | WT, WT, WT |
|  |  |  |  | A-19-2 | 64 | Smooth | 64, 32, 32 | C1496T, WT, WT |
| A-20 | *M. avium* | AMK (0.3) | 12/30/2010 | A-20-1 | 64 | Smooth | 64, >256, 64 | WT, A1408G, WT |
| A-21 | *M. avium* | SM (0.7) | 02/15/2011 | A-21-1 | 64 | Rough | 16, 16, 64 | WT, WT, WT |
|  |  |  |  |  |  | Smooth | 32, 32, 64 | WT, WT, WT |
|  |  |  | 02/09/2012 | A-21-2 | 64 | Smooth | 64, 32, 128 | WT, WT, C1496T |
| A-22 | *M. avium* | AMK (0.5) | 06/07/2012 | A-22-1 | 64 | Smooth | 32, >256, >256 | WT, A1408G, A1408G |
| A-23 | *M. avium* | AMK (6.9) | 03/06/2013 | A-23-1 | 64 | Smooth | 64, 64, 64 | WT, WT, WT |
| A-24 | *M. avium* | - | 07/15/2010 | A-24-1 | 64 | Smooth | 64, 64, 64 | WT, WT, WT |
|  |  |  | 01/20/2011 | A-24-2 | 64 | Smooth | 64, 64 | WT, WT |
|  |  |  | 11/15/2011 | A-24-3 | 64 | Smooth | 64, 64, 128 | WT, WT, A1408G |
|  |  |  | 01/17/2013 | A-24-4 | 64 | Smooth | 64, 32, 64 | WT, WT, WT |
|  |  |  | 08/27/2013 | A-24-5 | 64 | Smooth | 64, 64, 64 | WT, WT, WT |
|  |  |  | 04/17/2014 | A-24-6 | 64 | Smooth | 32, 32, 64 | WT, WT, WT |
| A-25 | *M. avium* | SM (0.5) | 02/25/2011 | A-25-1 | 128 | Smooth | 64, 64, 64 | WT, WT, WT |
|  |  | SM (5.4) | 08/11/2011 | A-25-2 | >128 | Smooth | 64, 64, 32 | WT, WT, WT |
|  |  | SM (9.3) | 12/08/2011 | A-25-3 | 64 | Smooth | 64, 64 | WT, WT |
|  |  | SM (12.8) | 07/10/2012 | A-25-4 | 64 | Smooth | 64, 64, >256 | WT, WT, A1408G |
|  |  | InhAMK (9.3) | 08/09/2016 | A-25-5 | >128 | Smooth | >256, >256, >256 | A1408G, A1408G, A1408G |
| I-1 | *M. intracellulare* | SM (3.1) | 09/10/2009 | I-1-1 | 64 | Smooth | 64, 64, 64 | WT, WT, WT |
|  |  |  |  | I-1-2 | 64 | Smooth | >256, 64, 64 | A1408G, WT, WT |
| I-2 | *M. intracellulare* | AMK (0.4), SM (3.0) | 10/18/2012 | I-2-1 | >128 | Smooth | 32, 32, 64 | WT, WT, WT |
| I-3 | *M. intracellulare* | SM (9.0) | 10/25/2011 | I-3-1 | 64 | Smooth | 64, 64, 32 | WT, WT, WT |
|  |  |  | 03/13/2012 | I-3-2 | >128 | Smooth | 8, 4, 4 | WT, WT, WT |
| I-4 | *M. intracellulare* | SM (3.2) | 10/21/2010 | I-4-1 | 64 | Smooth | 256, 64, 64 | C1409T, WT, WT |
| I-5 | *M. intracellulare* | SM (1.2) | 04/26/2011 | I-5-1 | 64 | Smooth | >256, 64, 32 | A1408G, WT, WT |
| I-6 | *M. intracellulare* | AMK (1.9) | 02/18/2013 | I-6-1 | 64 | Smooth | 64, 32, 32 | WT, WT, WT |
| I-7 | *M. intracellulare* | SM (12.3) | 08/31/2010 | I-7-1 | 64 | Smooth | 32, 32, 32 | WT, WT, WT |
|  |  | SM (15.4) | 09/27/2012 | I-7-2 | 64 | Smooth | 32, 64, 32 | WT, WT, WT |
| I-8 | *M. intracellulare* | SM (2.9) | 01/03/2013 | I-8-1 | 64 | Smooth | 64, 16, 32 | WT, WT, WT |
| I-10 | *M. intracellulare* | AMK (0.9) | 01/29/2009 | I-10-1 | 64 | Smooth | 32, 64, 64 | WT, WT, WT |
| I-12 | *M. intracellulare* | SM (2.3) | 09/27/2012 | I-12-1 | 64 | Smooth | 32, 32, 32 | WT, WT, WT |
|  |  | SM (7) | 02/14/2013 | I-12-2 | 64 | Smooth | 64, 64, 256 | WT, WT, G1491T |
| I-13 | *M. intracellulare* | SM (6.2) | 02/06/2013 | I-13-1 | 64 | Smooth | 64, 64, 128 | WT, WT, A1408G |
| I-14 | *M. intracellulare* | InhAMK (3.2) | 05/24/2016 | I-14-1 | 128 | Smooth | >256, >256, 256 | A1408G, A1408G, A1408G |
| I-16 | *M. intracellulare* | SM (4.7) | 02/08/2017 | I-16-1 | 64 | Smooth | 32, 32, >256 | WT, WT, A1408G |
| I-17 | *M. intracellulare* | SM (8.4) | 01/19/2017 | I-17-1 | 64 | Smooth | 64, 32, 64 | WT, WT, WT |
| I-18 | *M. intracellulare* | SM (3.0) | 07/30/2010 | I-18-1 | 64 | Smooth | 32, 64, 32 | WT, WT, WT |
| I-19 | *M. intracellulare* | AMK (2.0), SM (4.0) | 11/28/2013 | I-19-1 | >128 | Smooth | >256, >256 | A1408G, A1408G |
|  |  |  | 01/20/2015 | I-19-2 | >128 | Smooth | 64, >256, >256 | WT, A1408G, A1408G |
| I-20 | *M. intracellulare* | SM (2.3) | 10/31/2011 | I-20-1 | 64 | Smooth | 64, 64, >256 | WT, NA, A1408G |
| I-21 | *M. intracellulare* | SM (3.0) | 11/27/2012 | I-21-1 | 64 | Smooth | 32, >256, 32 | WT, A1408G, WT |
| I-23 | *M. intracellulare* | SM (7.6) | 11/25/2013 | I-23-1 | 64 | Smooth | 64, 64, 64 | WT, WT, WT |
| I-24 | *M. intracellulare* | SM (2.1) | 04/30/2016 | I-24-1 | 64 | Smooth | 32, 64, 64 | WT, WT, WT |
| I-25 | *M. chimaera* | SM (3.1) | 08/07/2013 | I-25-1 | 64 | Smooth | >256, >256 | A1408G, A1408G |
| I-26 | *M. intracellulare* | SM (4.4) | 01/15/2008 | I-26-1 | 64 | Smooth | 64, 64, 128 | WT, WT, A1408G |
|  |  |  | 08/04/2011 | I-26-2 | 128 | Smooth | 64, 64 | WT, WT |
|  |  |  | 04/12/2012 | I-26-3 | 64 | Smooth | 64, 64 | WT, WT |
|  |  |  | 09/27/2012 | I-26-4 | 64 | Smooth | 64, 256, 64 | WT, C1409T, WT |
| I-27 | *M. intracellulare* | SM (3.0), KM (2.5) | 08/31/2010 | I-27-1 | 64 | Smooth | 64, 64, 128 | WT, WT, G1491C |
| I-29 | *M. intracellulare* | SM (9.9) | 03/22/2012 | I-29-1 | 64 | Smooth | 64, 64, 64 | WT, WT, WT |
| I-30 | *M. intracellulare* | AMK (33.5), KM (21.3) | 07/12/2011 | I-30-1 | 64 | Smooth | 64, >256, 64 | WT, G1491T, WT |
| I-31 | *M. intracellulare* | SM (5.6) | 12/20/2012 | I-31-1 | 64 | Smooth | 64, 128, 64 | WT, A1408G, WT |
| I-32 | *M. intracellulare* | SM (6.1) | 08/09/2012 | I-32-1 | 64 | Smooth | 64, 32, 64 | WT, WT, WT |
|  |  | SM (11.9) | 01/31/2013 | I-32-2 | 64 | Smooth | 64, 64, 64 | WT, WT, WT |
|  |  | SM (17.4) | 07/16/2013 | I-32-3 | 128 | Smooth | 64, 64, 32 | WT, WT, WT |
|  |  | SM (18.7) | 01/23/2014 | I-32-4 | 64 | Smooth | 16, >256, 64 | WT, A1408G, WT |
| I-33 | *M. intracellulare* | SM (2.3) | 01/16/2013 | I-33-1 | 64 | Smooth | 64, 64, 32, >256 | WT, WT, WT, A1408G |
| I-34 | *M. intracellulare* | SM (1.3) | 09/01/2015 | I-34-1 | 64 | Smooth | 64, 64, 64 | WT, WT, WT |
| I-35 | *M. intracellulare* | - | 02/27/2014 | I-35-1 | 64 | Smooth | >256, 64 | A1408G, WT |
|  |  | SM (12.0) | 10/22/2015 | I-35-2 | 64 | Smooth | 64, 64, 64 | WT, WT, WT |
| I-36 | *M. intracellulare* | SM (24.3), KM (17.1) | 01/26/2016 | I-36-1 | >128 | Smooth | >256, >256, >256 | A1408G, A1408G, A1408G |
| I-37 | *M. intracellulare* | - | 09/12/2013 | I-37-1 | 64 | Rough | >256 | A1408G |
|  |  |  |  |  |  | Smooth | 32, 64, >256 | WT, WT, A1408G |
| I-38 | *M. intracellulare* | - | 11/07/2011 | I-38-1 | 64 | Smooth | 64, 64, 64 | WT, WT, WT |
|  |  |  | 11/07/2011 | I-38-2 | 64 | Smooth | 64, 32, 32 | WT, WT, WT |
| AB-7 | *M. abscessus* subsp. *abscessus* | AMK (0.4) | 10/19/2011 | AB-7-1 | 64 | Rough | 64, 64, 32 | C1409T, C1409T, WT |
| AB-9 | *M. abscessus* subsp. *abscessus* | AMK (5.7) | 06/01/2015 | AB-9-1 | 128 | Rough | >256, >256, >256 | A1408G, A1408G, A1408G |
|  |  |  | 01/19/2016 | AB-9-2 | 128 | Rough | >256, >256, >256 | A1408G, A1408G, A1408G |
|  |  | InhAMK (5.5) | 07/07/2016 | AB-9-3 | 128 | Rough | >256, >256, >256 | A1408G, A1408G, A1408G |
|  |  | InhAMK (12.0) | 01/19/2017 | AB-9-4 | 128 | Rough | >256, >256, >256 | A1408G, A1408G, A1408G |
| AB-13 | *M. abscessus* subsp. *abscessus* | AMK (1.9) | 07/03/2012 | AB-13-1 | 64 | Rough | 4, 4, 4 | WT, WT, WT |
|  |  |  |  |  |  | Smooth | >256, 4, >256 | A1408G, WT, A1408G |
| AB-15 | *M. abscessus* subsp. *abscessus* | AMK (2.1) | 10/25/2011 | AB-15-1 | 64 | Rough | 16, 16, 32 | WT, WT, WT |
|  |  |  |  |  |  | Smooth | 16, 256, 16 | WT, T1498A, WT |
| AB-17 | *M. abscessus* subsp. *abscessus* | - | 11/30/2010 | AB-17-1 | 64 | Rough | 4, 4, 8 | WT, WT, WT |
|  |  | AMK (0.9) | 10/04/2011 | AB-17-2 | 64 | Rough | 4, 4, 4 | WT, WT, WT |
|  |  |  |  |  |  | Smooth | 4, 4, 4 | WT, WT, WT |
|  |  |  | 01/22/2013 | AB-17-3 | >128 | Rough | 4, 4, 4 | WT, WT, WT |
|  |  |  |  |  |  | Smooth | 4, 4, 4 | WT, WT, WT |
|  |  |  | 10/21/2014 | AB-17-4 | 64 | Smooth | 64, 64, 64 | C1409T, C1409T, C1409T |
| M-1 | *M. abscessus* subsp. *massiliense* | AMK (0.9) | 09/02/2011 | M-1-1 | 64 | Rough | 16, 64 | WT, C1409T |
| M-2 | *M. abscessus* subsp. *massiliense* | AMK (0.9) | 11/06/2014 | M-2-1 | 64 | Smooth | >256, >256, >256 | A1408G, A1408G, A1408G |
|  |  |  | 02/05/2015 | M-2-2 | >128 | Smooth | >256, >256, >256 | A1408G, A1408G, A1408G |
|  |  |  | 08/04/2015 | M-2-3 | >128 | Rough | >256, >256, >256 | A1408G, A1408G, A1408G |
|  |  |  |  |  |  | Smooth | >256, >256, >256 | A1408G, A1408G, A1408G |
|  |  |  | 01/07/2016 | M-2-4 | >128 | Smooth | >256, >256, >256 | A1408G, A1408G, A1408G |
|  |  |  | 07/04/2017 | M-2-5 | >128 | Smooth | >256, >256, >256 | A1408G, A1408G, A1408G |
| M-3 | *M. abscessus* subsp. *massiliense* | AMK (5.8) | 04/02/2017 | M-3-1 | 64 | Rough | >256, >256, >256 | A1408G, A1408G, A1408G |

AMK: amikacin; InhAMK: inhaled amikacin; SM: streptomycin; KM: kanamycin; WT: wild-type; A: adenine; G: guanine; C: cytosine; T: thymine; NA: not available. ^*^ A total of 318 single colonies were obtained from 102 stored NTM clinical isolates.
